# Gastric and Rectal Metastases from Malignant Melanoma Presenting with Hypochromic Anemia and Treated with Immunotherapy

**DOI:** 10.1155/2017/2079068

**Published:** 2017-10-12

**Authors:** Pietro Genova, Maria Sorce, Daniela Cabibi, Gaspare Genova, Vittorio Gebbia, Daniela Galanti, Chiara Ancona, Maria Rosaria Valerio

**Affiliations:** ^1^Surgical Oncology Unit, Dipartimento di Scienze Chirurgiche, Oncologiche e Stomatologiche, University of Palermo, Palermo, Italy; ^2^Dermatology Unit, Dipartimento Biomedico di Medicina Interna e Specialistica (DiBiMIS), University of Palermo, Palermo, Italy; ^3^Pathology Unit, Dipartimento di Scienze Chirurgiche, Oncologiche e Stomatologiche, University of Palermo, Palermo, Italy; ^4^Medical Oncology Unit, La Maddalena Clinic for Cancer, Dipartimento Biomedico di Medicina Interna e Specialistica (DiBiMIS), University of Palermo, Palermo, Italy; ^5^Medical Oncology Unit, Dipartimento di Scienze Chirurgiche, Oncologiche e Stomatologiche, University of Palermo, Palermo, Italy

## Abstract

The authors present a case of an 80-year-old Caucasian male with multiple gastric and rectal metastases from malignant melanoma presenting with hypochromic anemia as the sole symptom of disease without evidence of cutaneous and ocular tumor localization. The patient had a medical history positive for malignant lentigo melanoma of the occipital region of the scalp and early stage laryngeal squamous cell carcinoma and prostatic carcinoma treated with radiation therapy. The authors make some considerations on intestinal involvement by metastatic melanoma and discuss the choice of not treating with endoscopic procedures the gastric metastatic lesions most likely responsible for the clinical sign present at diagnosis. The patient was referred to clinical oncologists and received immunotherapy with ipilimumab and pembrolizumab.

## 1. Introduction

Metastatic involvement of the gastrointestinal tract by epithelial malignancies is a rather infrequent event as opposed to malignant melanoma, which appears to have a particular trend towards metastasis to the gut [1]. In fact, autopsy series report up to 60% pathological intestinal involvement in patients that deceased once diagnosed with metastatic melanoma, which however reaches clinical relevance in a median of 2% of patients only (range: 0.8–8.9%) [2, 3]. The wide range of incidences can be partly explained by difficulties in exploring the small intestine, which usually represents the most frequent localization of gastrointestinal metastasis, as well as the absence of specific symptoms which are more frequently reported in gastric and duodenal localization more easily accessible for diagnostic study [4].

## 2. Objective

The objective of this paper is to present a case of an unusual clinical presentation of metastatic melanoma with hypochromic anemia refractory to iron therapy treated with endoscopic procedures and systemic immunotherapy. Review of the literature is also reported below.

## 3. Case Report

In 2004, an 80-year-old Caucasian male underwent bilateral cordectomy for Tis carcinoma in the left cord and microinvasive T1 carcinoma at the right one. In the same year, he was diagnosed with prostatic carcinoma (Gleason score 3 + 3) which was treated with radiation therapy for a final total dose of 64 Gy. Since then, he has been controlled regularly on a 6-month basis. His family history was negative for cancer, but he suffered from mild hypertension that has been treated with a sartan drug for 17 years, and he also suffered from mild dyslipidemia without any medication besides dietary control.

In January 2014, he was admitted at the dermatologic unit of our institution for a pigmented cutaneous lesion at the occipital region of the scalp and an ulcerated lesion in the left temporal region, which were both radically excised. The pathologist reported a BRAF, c-KIT, and N-RAS wild-type* lentigo maligna* melanoma in the occipital scalp, while the ulcerated lesion at the temporal region was classified as an actinic keratosis. Full skin and ocular examinations, chest X-rays, and abdominal sonogram excluded the presence of clinical detectable neoplastic deposits. Due to his medical history, the patient was admitted to a three-month follow-up program. In January 2015, for the appearance of a swelling in the left parotid gland, he received an MRI and a subsequent biopsy, which led to a diagnosis of pleomorphic adenoma.

**Figure 1:**
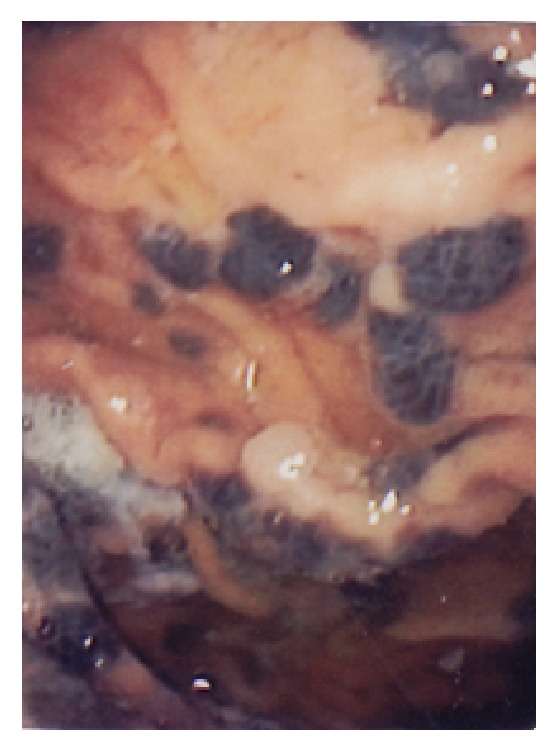
Presence of several medium size melanotic lesions in the fundus and the body of the stomach.

**Figure 2:**
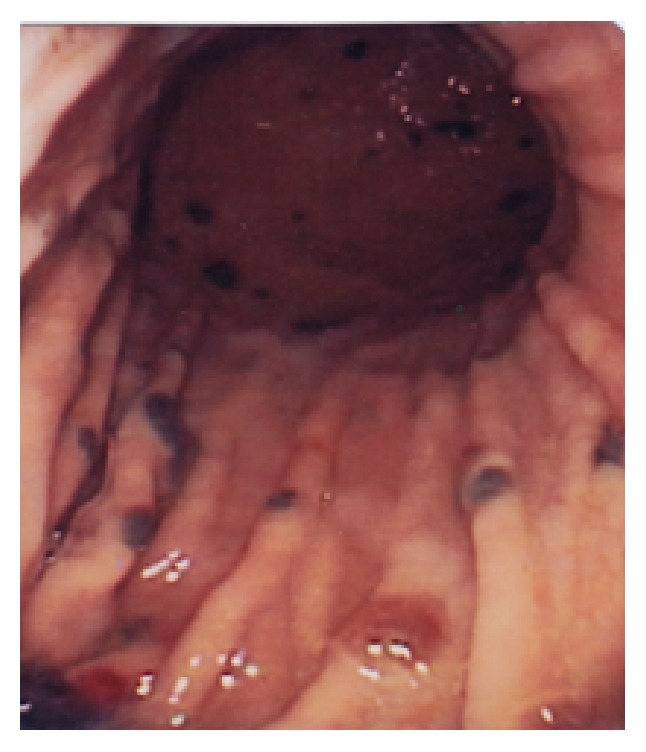
Melanotic lesions are less frequent in the distal part of the stomach.

In May 2014, he was diagnosed with hypochromic, iron-deficiency anemia in the absence of overt clinical signs of bleeding and normal urine analysis. Fecal occult blood test was strongly positive. The patient was put on oral iron therapy without positive results for three months and therefore was referred to the endoscopy unit for upper and distal gastrointestinal tracts examination. Esophagogastroduodenoscopy showed the presence of several nonbleeding, bluish to black, gastric lesions of a few millimeters in diameter (Figures 1 and 2). Such lesions were present in the whole stomach with an increased frequency in the corpus and the antrum but were absent in the esophageal tract and the duodenal level until Treitz's ligament. Biopsy of the three lesions was performed without significant bleeding. Morphologically, the lesions appeared suggestive for metastatic melanoma. Pan-colonoscopy was performed two days later and showed no widespread injuries except for two 5 mm wide, brownish, nonbleeding areas 2 cm away from each other at the rectal mucosa about 15 cm from the anal verge. Biopsy was suddenly performed although the morphological features of these rectal lesions were consistent with mucosal angiodysplastic lesions similar to those often found after radiation therapy for prostate tumors. Pathology examination of both gastric and rectal specimens reported the presence of HMB45, MelanA-S100+, Cd68 negative, infiltrating malignant melanoma. PET/TC scan showed a metastatic lesion in the lumbar spine and the left clavicle, with the latter being biopsied. Pathology reported metastatic melanoma to the bone. PSA and serum chemistry tests were within the normal limits. Careful skin mapping as well ocular examination failed to show presence of cutaneous melanoma. In April 2015, rectal lesions were endoscopically treated with argon plasma coagulation, while gastric lesions were left untreated. The patient was subsequently referred to the Medical Oncology Unit and, after we obtained written informed consent, we started immunotherapy with ipilimumab 3 mg/kg every three weeks. After 3 months, he showed rapidly progressing disease with the appearance of melena and worsening of anemia. Therefore, ipilimumab was stopped and in July 2015 pembrolizumab was started at the dose of 2 mg/kg every three weeks. Reevaluation of the disease after three months showed a PET/TC-documented stabilization of the disease and a significant improvement of clinical conditions and hemoglobin levels with a dramatic reduction of transfusion needs. Pembrolizumab was very well tolerated with mild gastrointestinal and cutaneous toxicity, easily managed with low dose steroids. Pembrolizumab was given for eight months till March 2016 when progressive disease to the liver and the brain was recorded. Unfortunately, the patient died 4 months later due to massive liver progression.

## 4. Considerations

Primary malignant gastrointestinal melanoma may arise in several areas of the digestive tract: 33% of the cases are located in the nasopharynx, 5.9% in the esophagus, 2.7% in the stomach, 2.3% in the small intestine, 1.4% in the gallbladder, 9% in the colon, 22% in the rectum, and 31% in the anal tract [1–6]. On the other hand, primary cutaneous melanoma with gastrointestinal metastasis may be localized in the head and neck region (3–33%), being much more frequent in the limbs (15–57%) and the trunk (13–54%) [1–6]. Overall, 20% of cases with gastrointestinal metastasis from melanoma present gastric metastases, 58% small intestine lesions, and 22% colorectal lesions. Cutaneous melanoma has a distinct pattern of metastasis, preferentially targeting the submucosa of the small intestine probably via the CCR9 lymphocytes, CCL25 thymus-expressed chemokine axis, and specific integrins [7]. The incidence of intestinal metastasis is also related to Clark level of the primary lesion, being less than 6% for level I, 6 to 24% for level II, and more than 70% for level III or greater.

Endoscopically, gastric metastasis from melanoma may appear as black-pigmented ulcers, diffuse black pigment in the context of mucosa, multiple small size nodules of the mucosa or submucosa, polypoid lesions, or extrinsic masses [8]. These lesions are often pigmented but may be nonpigmented, mimicking other forms of neoplastic epithelial lesions or MALT lymphomas [1–6]. However, the majority of patients with gastrointestinal metastatic spread from malignant melanoma present multiple metastases localized to the small intestine with a wide morphological variability [9]. Symptoms are often modest and rather nonspecific, and clinical indication for endoscopic study stems only from the need to find the cause of occult bleeding responsible for chronic, iron-deficiency anemia as in this patient [4]. Unfortunately, intestinal metastases from melanoma may lead to nonspecific surgical and medical emergency conditions such as bowel obstruction and/or perforation, dyspepsia, nausea and vomiting, abdominal pain, weight loss, diarrhea, and overt bleeding [10]. More rarely, metastatic deposits may cause, as in the present case, chronic hypochromic anemia. Clinically silent gastrointestinal metastases can be present in an increasing percentage of cases, therefore rendering it reasonable to include endoscopy in follow-up exams of selected patients with mild gastroenteric symptoms [4]. Biopsy during the endoscopic procedure is mandatory since many polypoid lesions can be nonmelanotic. For this reason, in patients with a history of melanoma in which endoscopic polypoid lesion is observed, the presence of a nonpigmented lesion is a plausible suspicion of a possible metastatic lesion. The soft capsule study of the ileum, despite its limitations, could provide further data due to the higher frequency of metastasis localization in this intestinal tract [11]. PET/CT has a very high sensitivity in detecting metastasis from melanoma [12, 13]. In our case, the PET-CT showed vertebral and clavicular metastases, which however were biopsied to rule out metastatic deposits from other neoplasms since medical history was positive for prostate and larynx cancer. However, PET/TC was not able to show metastatic lesions or gastric lesions. In such cases, the role of surgery is palliative [14]. Surgical interventions for symptomatic patients with melanoma of the gastrointestinal tract significantly relieve pain and improve quality of life and may confer a survival advantage.

Medical oncologists choose systemic immunotherapy with the monoclonal anti-CTLA4 antibody ipilimumab, which has been proven to be effective in the treatment of metastatic melanoma. In fact, ipilimumab, with or without a gp100 peptide vaccine, as compared with gp100 alone, improved overall survival in patients with previously treated metastatic melanoma [15]. The median overall survival was 10.0 months among patients receiving ipilimumab plus gp100, as compared with 6.4 months among patients receiving gp100 alone (hazard ratio for death: 0.68; *P* < 0.001). The median overall survival with ipilimumab alone was 10.1 months (hazard ratio for death in comparison with gp100 alone: 0.66; *P* = 0.003). No difference in overall survival was detected between the ipilimumab groups (hazard ratio with ipilimumab plus gp100: 1.04; *P* = 0.76). At progression after ipilimumab treatment, the patient was offered to start second-line therapy with the anti-PD-1 monoclonal antibody pembrolizumab according to the results of the Keynote-002 trial [16]. This phase II study compared two different doses (2 versus 10 mg/kg every three weeks) of pembrolizumab versus investigator-choice chemotherapy (paclitaxel plus carboplatin, paclitaxel, carboplatin, dacarbazine, or oral temozolomide) in a series of patients with metastatic melanoma pretreated with ipilimumab. Both dosages of pembrolizumab were superior to chemotherapy in terms of progression-free survival (5.5 months versus 5.8 versus 3.6 months, resp.). A subsequent phase III clinical trial showed that pembrolizumab was more active than ipilimumab in terms of PFS. The estimated 6-month progression-free survival rates were 47.3% for patients receiving pembrolizumab every 2 weeks, 46.4% for those receiving pembrolizumab every 3 weeks, and 26.5% for those receiving ipilimumab. Median estimates of progression-free survival were 5.5 months, 4.1 months, and 2.8 months, respectively. A recent review comprising all open-label studies with pembrolizumab reported a 33% overall response rate with a rate of 35% a year equal to the PFS and finally an overall median survival of 23 months [17]. Only 14% of patients treated with pembrolizumab developed grade 3 toxicity. The patient experienced an eight-month progression-free survival, longer than median PFS reported for pembrolizumab, with an excellent tolerability that enabled him to maintain a good quality of life with optimal symptom control.

## 5. Conclusions

This case is intriguing for both clinical presentation and treatment challenges. In fact, occult gastrointestinal bleeding as the first clinical presentation of metastatic melanoma is relatively unusual, especially in the absence of other symptoms of disease. Gastrointestinal metastasis, especially of the small intestine, always represents an advanced stage of the disease even in the absence of other evidence of disease and should be always suspected in patients with gastrointestinal symptoms and history of cutaneous melanoma. The massive involvement of the stomach and the coexistence of rectal lesions as well as the simultaneous presence of bone metastasis are suggestive for metastatic spread rather than a primitive gastrointestinal melanoma. Therefore, after intensive workup, we concluded that gastrointestinal metastatic lesions detected in this case may be secondary to the excised malignant lentigo at the occipital region of the scalp even if this neoplastic malignant disease is reported to have a low metastatic potential.
