# Erratum to “Gastric and Rectal Metastases from Malignant Melanoma Presenting with Hypochromic Anemia and Treated with Immunotherapy”

**DOI:** 10.1155/2018/3142394

**Published:** 2018-02-27

**Authors:** Pietro Genova, Maria Sorce, Daniela Cabibi, Gaspare Genova, Vittorio Gebbia, Daniele Galanti, Chiara Ancona, Mara Rosaria Valerio

**Affiliations:** ^1^Surgical Oncology Unit, Dipartimento di Scienze Chirurgiche, Oncologiche e Stomatologiche, University of Palermo, Palermo, Italy; ^2^Dermatology Unit, Dipartimento Biomedico di Medicina Interna e Specialistica (DiBiMIS), University of Palermo, Palermo, Italy; ^3^Pathology Unit, Dipartimento di Scienze Chirurgiche, Oncologiche e Stomatologiche, University of Palermo, Palermo, Italy; ^4^Medical Oncology Unit, La Maddalena Clinic for Cancer, Dipartimento Biomedico di Medicina Interna e Specialistica (DiBiMIS), University of Palermo, Palermo, Italy; ^5^Medical Oncology Unit, Dipartimento di Scienze Chirurgiche, Oncologiche e Stomatologiche, University of Palermo, Palermo, Italy

In the article titled “Gastric and Rectal Metastases from Malignant Melanoma Presenting with Hypochromic Anemia and Treated with Immunotherapy” [1], the name of the sixth author was given incorrectly as Daniela Galanti. The author's name should have been written as Daniele Galanti. The revised authors' list is shown above.
